# Supplementary material for: Sustained-input switches for transcription factors and microRNAs are central building blocks of eukaryotic gene circuits
Source: Genome Biol. 2013 Aug 23;14(8):R85. doi: 10.1186/gb-2013-14-8-r85 (PMC4054853; doi:10.1186/gb-2013-14-8-r85)
Supplement: Additional file 5 — HTML Browsable Motif Output. Zipped folder containing all WaRSwap and FANMOD motif output, viewable in a web browser. [file gb-2013-14-8-r85-S5.ZIP › HTML_browsable_motif_output/WaRSwap_ath_tair9/sigs_WSWRSLDraw_2-2000.pvals.heatmaps.html/motif_id_36_001011002_tftype_ath_upstream_-2000_0.html]

```
BG_MODEL = Rnew2
MOTIF_ID = 36_001011002
TF_TYPE = ath
UPSTREAM = -2000_0


PVals
FN_0.2	FN_0.4	FN_0.6	FN_0.8
dg_60.genes	0.004	0.03	0.777	1
dg_70.genes	0.004	0.048	0.738	1
dg_80.genes	0.022	0.028	0.645	1

ZScores
FN_0.2	FN_0.4	FN_0.6	FN_0.8
dg_60.genes	2.64	2.005	-0.941	-7.687
dg_70.genes	2.591	1.811	-0.747	-7.176
dg_80.genes	2.662	2.082	-0.342	-5.926

StDevs
FN_0.2	FN_0.4	FN_0.6	FN_0.8
dg_60.genes	3379.833	1870.543	760.391	405.021
dg_70.genes	2494.676	1422.563	556.505	301.946
dg_80.genes	1063.045	610.988	285.27	175.196
```
